# Effective *Agrobacterium*-Mediated Transformation System for Eureka Lemon Using Whole Cotyledonary Node

**DOI:** 10.3390/plants14111629

**Published:** 2025-05-27

**Authors:** Jinfa Zhao, Yuan Chen, Jiajun Wang, Chunqing Wang, Yan Zhou

**Affiliations:** 1 Integrative Science Center of Germplasm Creation in Western China (Chongqing), Science City/Citrus Research Institute, Southwest University, Chongqing 400712, China; 15779738537@163.com (J.Z.); 18278207783@163.com (Y.C.); 13251317657@163.com (J.W.); 2 Institute of Plant Protection, Jiangxi Academy of Agricultural Sciences, Nanchang 330200, China; 15707972785@163.com

**Keywords:** lemon transformation, whole cotyledonary node, explant, transformation efficiency

## Abstract

*Agrobacterium*-mediated transformation systems using epicotyl explants have been widely used for genetic transformations of citrus. However, their application in lemons is severely constrained by browning of epicotyl tissues, which leads to an extremely low efficiency of transformation. In this study, we developed an optimized *Agrobacterium*-mediated transformation system using whole cotyledonary node explants of ‘Eureka’ lemon (*Citrus limon*), which significantly reduced tissue browning and enhanced transformation efficiency up to 14.48%. In addition, preparation of the whole cotyledonary node was simple and rapid, which reduced time and labor. This system facilitated efficient generation of transgenic lemon plantlets and provided a novel explant source for citrus transformation.

## 1. Introduction

Citrus is an economically important fruit crop, which is widely cultivated in tropical and subtropical regions worldwide. By 2021, global citrus production will reach 161.801 million tons, with more than 10.2 million hectares under cultivation [1].

Citrus cultivation faces persistent challenges from multiple pathogens, resulting in significant yield losses [2,3,4]. Given the limited availability of chemical control agents, breeding for disease resistance has become a critical strategy for sustainable management of Huanglongbing and viral diseases, including citrus tristeza virus, citrus yellow vein clearing virus, and citrus chlorotic dwarf-associated virus [5,6,7].

In addition to selecting bud sports, conventional and somatic hybridization and *Agrobacterium*-mediated gene transformation technology have been widely used in citrus disease resistance breeding, and some citrus (e.g., *Citrus sinensis*, *Carrizo citrange*, and *Citrus aurantifolia*) germplasms that are resistant to citrus canker and Huanglongbing have been obtained [8,9,10].

Various citrus explants have been used for genetic transformation [11,12]. Among them, cultivation of protoplasts and embryonic calli requires advanced technical expertise and is highly challenging [13,14]. In addition, cotyledon and leaf explants have reported or good regenerative transformation efficiency only in certain citrus cultivars [15,16]. Currently, juvenile epicotyls are considered relatively highly effective and popular explants for citrus transformation [17]. However, the internodal stems or epicotyls of lemons are susceptible to tissue browning, resulting in low shoot regeneration and difficulty in obtaining transgenic lemons [18,19]. Unfortunately, reports of lemon transformation using other explants are scant.

Cotyledons and whole cotyledonary nodes have been widely used as preferred explant sources for studies on genetic transformation in annual plants, such as cotton (*Gossypium* spp.), cucumber (*Cucumis sativus* L.), soybean (*Glycine max* L.), *Paeonia lactiflora Pall*, *Malus micromalus*, and *Lawsonia inermis* L., owing to their advantages of a high and rapid regeneration frequency, homogeneous genetic background, and easy processing [20,21,22,23,24,25]. To date, no reports on the genetic transformation of lemons using cotyledonary nodes are available.

Therefore, in the present study, we aimed to establish an *Agrobacterium*-mediated transformation system using the whole cotyledonary node of Eureka lemon (*Citrus limon*) to obtain transgenic lemons and improve the efficiency of genetic transformation in Eureka lemons.

## 2. Results

### 2.1. Epicotyls of Eureka Lemon Are Highly Recalcitrant to Agrobacterium-Mediated Transformation

**Figure 1:**
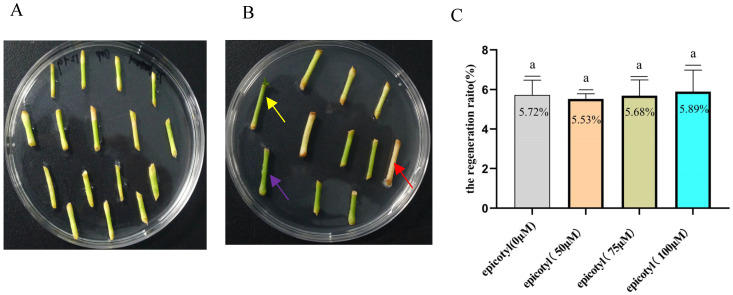
The regeneration ratio of Eureka lemon epicotyls. (**A**) Eureka lemon epicotyls on day 10 after culture in shoot regeneration medium (SRM). (**B**) Eureka lemon epicotyls exhibiting severe browning on day 60 after culture in SRM. The epicotyl indicated by the red arrow exhibits severe browning throughout the whole tissue. The epicotyl indicated by the purple arrow exhibits severe browning at the cut end. The epicotyl indicated by the yellow arrow developed a weak shoot. (**C**) The regeneration ratio of epicotyls on day 60 after culture in SRM supplemented with different concentrations of melatonin (0, 50, 75, and 100 μM). The same lowercase letters indicate no significant differences at *p* < 0.05 level (Tukey’s HSD).

We genetically transformed Eureka lemon epicotyls to determine their regeneration and transformation efficiencies. A total of 613 epicotyls were used for transformation (Supplementary Table S1). Ten days after culturing in SRM, most epicotyls exhibited browning, which eventually led to necrosis and failure to produce shoots (Figure 1A,B). By day 60, only a few Eureka lemon epicotyls had developed weak shoots (Figure 1B), yielding a low regeneration ratio of 5.72% (Figure 1C). Transgenic shoots were not observed. In addition, melatonin failed to inhibit browning, and no significant improvement in the regeneration rate was observed compared with that of the untreated control (Figure 1C).

### 2.2. Whole Cotyledonary Node Explants from Eureka Lemon Exhibited Higher Transformation Efficiency than Epicotyl Explant

**Figure 2:**
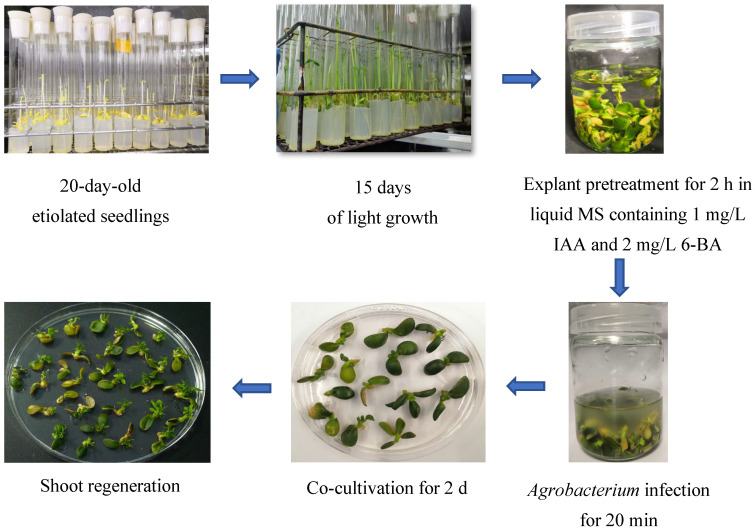
Schematic of the procedure for *Agrobacterium*-mediated transformation of citrus.

**Figure 3:**
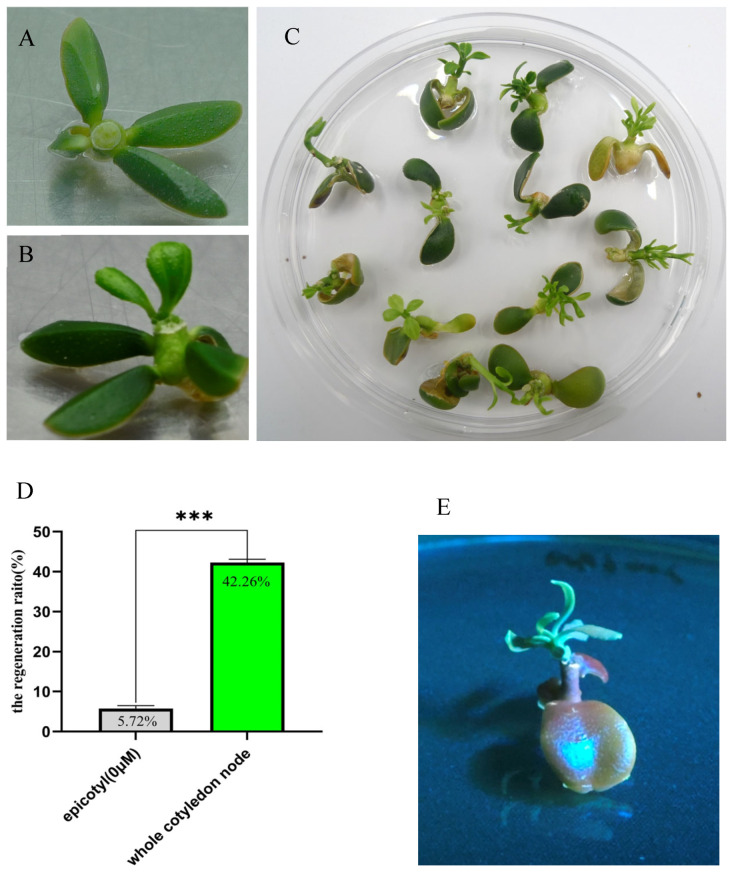
Shoot regeneration of Eureka lemon whole cotyledonary nodes in shoot regeneration media (SRM). (**A**) Whole cotyledonary node on day 10 after culture in SRM. (**B**) Whole cotyledonary node on day 15 after culture in SRM. (**C**) Whole cotyledonary nodes on day 60 after culture in SRM. (**D**) Comparison of regeneration ratios of epicotyls and whole cotyledonary nodes. *** *p* < 0.01 (*t*-test). (**E**) Transgenic shoot on whole cotyledonary node exhibiting green fluorescent protein signal under ultraviolet light.

To improve the genetic transformation efficiency of Eureka lemons, cotyledonary nodes were used as explants for genetic transformation. (The procedure for transformation is shown in Figure 2) A total of 142 whole cotyledonary nodes were used for transformation (Supplementary Table S1). After 10 days, half of the whole cotyledonary nodes grew normally and produced calli at the wound sites (Figure 3A). After 15 days, the calli from the whole cotyledonary nodes divided and produced vigorous fascicular shoots (Figure 3B). A high regeneration rate of 42.26% was observed after 60 days (Figure 3C,D). The regeneration rate of whole cotyledonary nodes was approximately eight times higher than that of the epicotyl. Nine shoots (14.48%) exhibited GFP fluorescence in all tissues under ultraviolet light (Figure 3E). These results demonstrated the high efficiency of using whole cotyledonary nodes of Eureka lemons as explants for genetic transformation.

### 2.3. PCR Identification of Transgenic Plants

**Figure 4:**
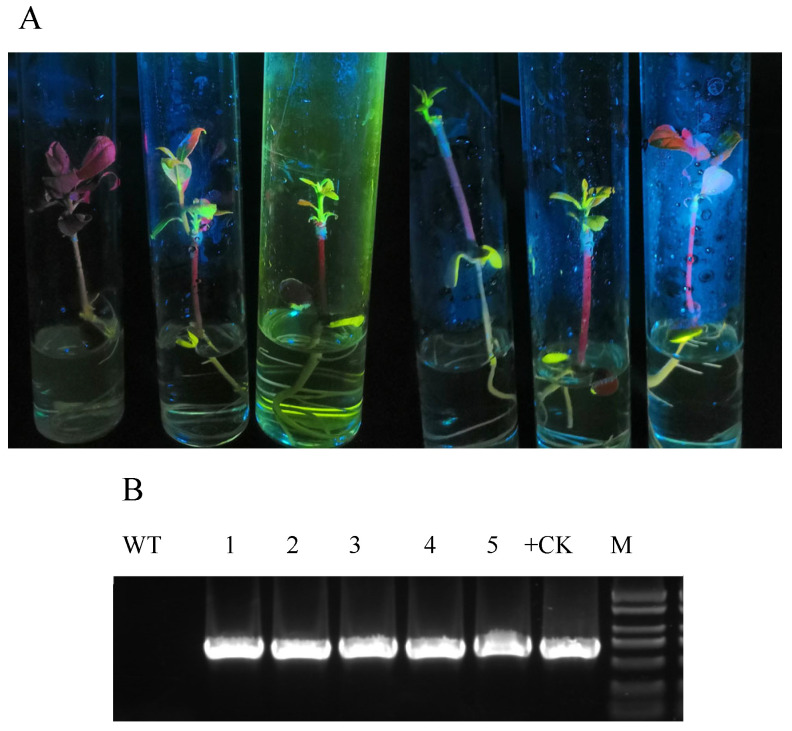
Identification of transgenic plants. (**A**) Transgenic Eureka lemon seedlings exhibiting green fluorescent protein (GFP) signal under ultraviolet light. (**B**) Amplification of genomic DNA of transgenic plants by polymerase chain reaction. WT, wild-type plant; 1–5, GFP-expressing plants; +CK, positive control using pNmGFPer plasmid DNA as template.

To determine stable GFP expression, transgenic shoots were grafted onto 10-day-old etiolated Eureka lemon seedlings and monitored. Green fluorescence was always observed in all five seedlings (Figure 4A). Additionally, PCR analysis confirmed that *gfp* was integrated into the Eureka lemon genome (Figure 4B). These results demonstrated that the cotyledonary nodes of Eureka lemons used as explants for genetic transformation were stable.

Overall, these results demonstrated that the *Agrobacterium*-mediated transformation system using whole cotyledonary nodes significantly enhanced the morphogenesis and regeneration ratio of Eureka lemon calli, and successfully generated transgenic plants.

## 3. Discussion

Various citrus explants have been used in genetic transformation studies, with the epicotyl segments being most widely used. High transformation efficiencies have been reported in multiple citrus cultivars, including *Citrus sinensis*, *Carrizo citrange*, sour orange (*Citrus aurantium*), and grapefruit (*Citrus paradisi*), when epicotyl explants are used [26,27,28,29]. However, our study revealed that epicotyls of Eureka lemons were recalcitrant to *Agrobacterium*-mediated transformation. This recalcitrance was characterized by extensive tissue browning and a low regeneration rate, ultimately hindering the recovery of transgenic lines. Although melatonin, a known antioxidant, was used to mitigate browning, its efficacy in this system was insufficient. These findings underscore the necessity of improving the methodology for establishing an efficient genetic transformation protocol for Eureka lemons.

The whole cotyledonary node, known for its high regenerative capacity and rapid production of adventitious shoots, has emerged as an excellent source of explants for genetic transformation in various plant species [20,21,22,23,24,25]. Furthermore, its accessibility and structural simplicity significantly reduce the time and labor required for explant preparation compared to that for traditional epicotyl-based systems [24]. In the present study, we developed an optimized *Agrobacterium*-mediated transformation protocol using whole cotyledonary node explants from Eureka lemon. The whole cotyledonary node system shortened the operational time and reduced labor requirements. Notably, the whole cotyledonary node system exhibited enhanced tolerance to oxidative tissue browning and achieved a high transformation efficiency.

The severity of explant browning is strongly associated with the accumulation of phenolic compounds and cytotoxic quinones at elevated levels, which impair cell viability and the capacity of callus differentiation [19,30]. Previous studies on Citrus species, such as Mexican lime (*Citrus aurantifolia*) and Carrizo citrange, have reported that antioxidant supplementation during in vitro culture effectively inhibits oxidative browning and enhances the regeneration efficiency of epicotyl explants [27,31]. However, in this study, melatonin treatment did not suppress browning or improve the shoot regeneration ratio of Eureka epicotyls, probably owing to differences in the levels of phenolic compounds between Eureka lemon, Mexican lime, and Carrizo citrange. Eureka lemon epicotyls potentially synthesize polyphenolic compounds or quinones at relatively high levels where there are wounds, *Agrobacterium* stress, or stimulation by regulatory factors in the medium, compared to those by other citrus cultivars. In contrast, whole cotyledonary nodes suffer relatively low *Agrobacterium* stress and stimulation by regulatory factors, owing to most tissue being outside the medium. This may explain relatively low browning and high regeneration in whole cotyledonary nodes. In addition, previous study also suggested that higher regeneration and transformation rates can be achieved for light-grown seedlings than for etiolated seedlings [27]. Explant- or citrus-specific stress responses to environmental factors, such as light conditions, growth regulators, and nutrient solutions, may affect explant browning.

Beyond explant selection, critical factors that influence the success of transformation include concentration of the *Agrobacterium* strain, types of antioxidants, types and ratios of growth regulators (e.g., nutrient solutions and phytohormones) in the culture medium, and vector design [12]. Several studies have focused on optimizing these parameters for enhancing transformation efficiency [19,26,27,28]. Future studies should focus on optimizing these transformation parameters for maximizing efficiency and assessing compatibility with diverse vector constructs and target genes. In addition, the protocol should be extended to other commercially relevant citrus genotypes. These efforts may strengthen the robustness and applicability of this system for large-scale genetic improvement programs in citrus.

## 4. Materials and Methods

### 4.1. Vector and Medium

The pNmGFPer plasmid containing a 2 × 35S promoter-driven enhanced green fluorescent protein (eGFP) reporter gene cassette with a nopaline synthase terminator (NOS-ter) was used for *Agrobacterium tumefaciens*-mediated transformation of Eureka lemon. The co-cultivation medium (CM) contained Murashige and Skoog Medium (MS) with modified base salts and vitamins (Coolaber, Beijing, China), 3% (*w*/*v*) sucrose, 1 mg/L indole-3-acetic acid, 1 mg/L 6-dimethylallylamino purine riboside, 1 mg/L 2,4-dichlorophenoxy acetic acid, and 0.1 M/L acetosyringone (pH, 5.8). The shoot regeneration medium (SRM) contained MS with modified base salts and vitamins, 3% (*w*/*v*) sucrose, 0.5 mg/L indole-3-acetic acid, 1 mg/L 6-dimethylallylamino purine riboside, 2.5 mg/L N-(phenylmethyl)-9H-purin-6-amine, 500 mg/L cefotaxime, and 75 mg/L kanamycin (pH, 5.8). The explant pretreatment solution contained MS with modified base salts and vitamins, 3% (*w*/*v*) sucrose, 1 mg/L indole-3-acetic acid, and 2 mg/L N-(phenylmethyl)-9H-purin-6-amine (pH, 5.8). The resuspension solution contained MS with modified base salts and vitamins, and 3% (*w*/*v*) sucrose (pH, 5.4).

### 4.2. Plant Material and Explant Preparations

Mature Eureka lemon seeds were collected from AnYue, Chongqing, China in 2024, and the experiment was carried out between June and December in 2024. Eureka lemons were genetically transformed as previously described [32]. After removing seed coats, 100 Eureka lemon seeds were sterilized with 5% sodium dichloroisocyanurate for 10 min. Subsequently, seeds were washed 10 times with 1 L sterile water and then cultured on MS modified medium with vitamins, sucrose, and agar (Coolaber, Beijing, China) in a dark plant culture chamber at 28 °C. After 20 days, etiolated seedlings were transferred to a plant culture chamber at 28 °C under a 16/8 h day/night cycle for 15 days until the cotyledons and epicotyls turned completely green. Whole cotyledonary node explants were obtained by removing roots of seedlings and retaining approximately 0.5 cm epicotyl and two cotyledons [23]. For epicotyl explants, epicotyls were cut into approximately 2 cm pieces. Whole cotyledonary nodes and epicotyl segments were immersed in the pretreatment solution for 2 h.

### 4.3. Agrobacterium Preparation

*Agrobacterium* EHA105 harboring pNmGFPer was cultured in Luria–Bertani medium (LB) containing 20 μg/L rifampicin and 50 μg/L kanamycin at 28 °C for 1 day. Subsequently, the *Agrobacterium* culture was inoculated into fresh LB medium containing 20 μg/L rifampicin and 50 μg/L kanamycin. The culture was shaken for 8 h until the optical density at 600 nm (OD_600_) reached 0.8. The culture was centrifuged, and *Agrobacterium* was resuspended in resuspension solution to a final concentration of OD_600_ = 0.8.

### 4.4. Co-Cultivation and Shoot Regeneration

Explants were soaked in *Agrobacterium* suspension for 20 min and then dried using sterile filter paper. Subsequently, both explants were placed on CM and incubated in a dark plant culture chamber at 28 °C. After 2 days, whole cotyledonary nodes were transferred to SRM. Previous studies have shown that antioxidant treatment can inhibit epicotyl browning and improve regeneration efficiency [19]. To investigate the inhibitory effect of melatonin on epicotyls browning, epicotyls were transferred into SRM supplemented with different concentrations of melatonin (0, 50, 75, and 100 μM/L). Both explants were cultured in the dark at 28 °C for 7 days and were subsequently subjected to a 16 h photoperiod for 2 months.

### 4.5. Micrografting and Identification of Transgenic Plants by Polymerase Chain Reaction (PCR)

Micrografting was performed as previously described [26]. Briefly, shoots were beveled into a V-shaped notch at the shoot tip and were then grafted individually onto 10-day-old etiolated Eureka lemon seedlings. Grafted seedlings were transferred into MS liquid medium and grown in a plant culture chamber at 28 °C. After 2 months, total DNA was extracted from newly emerging leaves of transgenic plants using a DNA extraction kit (CWBIO, Beijing, China). PCR was performed to detect *gfp* using the primers GFP-F/GFP-R: 5′-cctcggccgaattcagtaaa-3′ and 5′-gggcagattgtgtggacag-3′. The PCR system comprised 5 μL Green Taq Mix (Vazyme, Nanjing, China), 1 μL DNA, and 200 nM each of the forward and reverse primers, and 3.6 μL enzyme-free water. The reaction conditions were 34 cycles of 20 s at 95 °C, 15 s at 55 °C, and 30 s at 72 °C. The amplified products were purified using an EasyPure Quick Gel Extraction Kit (TransGen, Beijing, China) and sequencing.

### 4.6. Data Analysis

All experiments were repeated twice. The regeneration efficiency was calculated as the ratio of germinated explants to the total number of explants. Transgenic shoots were identified by observing green fluorescent protein (GFP) expression. Transformation efficiency was defined as the number of GFP-positive shoots divided by the total number of germinated explants [19].

The data were analyzed using GraphPad Prism v.9.3.1. The Honestly Significant Difference test was used to assess differences among ≥3 groups. A *t*-test was used to analyze the differences between two groups. Data are expressed as mean ± standard deviation (SD).

## 5. Conclusions

We established an optimized *Agrobacterium*-mediated transformation system utilizing whole cotyledonary node explants of Eureka lemon. This study will contribute to efficient breeding for generating disease-resistant lemon varieties.

## Data Availability

Data are contained within the article.

## Supplementary Materials

The following supporting information can be downloaded at https://www.mdpi.com/article/10.3390/plants14111629/s1, Table S1: Statistics on the regeneration rate and transformation efficiency of explants
