# Supplementary material for: Effective Agrobacterium-Mediated Transformation System for Eureka Lemon Using Whole Cotyledonary Node
Source: Plants (Basel). 2025 May 27;14(11):1629. doi: 10.3390/plants14111629 (PMC12157941; doi:10.3390/plants14111629)
Supplement: Supplementary file 1 [file plants-14-01629-s001.zip › plants-3639260-supplementary.pdf]

Table S1 Statistics on the regeneration rate and transformation efficiency of explants

Explant type	Number of explants	concentration of melatonin-supplemented SRM	Number of regeneration explants	regeneration ratio	Number of positive explants	transformation efficiency
Epicotyl	77	0 μM	4	5.19% (4/77)	0	0
	80		5	6.25% (5/80)	0	0
	75	50μM	4	5.33% (4/75)	0	0
	70		4	5.71% (4/70)	0	0
	78	75 μM	4	5.12% (4/78)	0	0
	80		5	6.25% (5/80)	0	0
	75	100 μM	5	6.66% (5/75)	0	0
	78		4	5.12% (4/78)	0	0
Whole cotyledonary nod	72	0 μM	30	41.66% (30/72)	4	13.33% (4/30)
	70		32	42.85% (32/70)	5	15.62% (5/32)

Note: SRM, shoot regeneration medium.
